# Supplementary material for: An arithmetic method algorithm optimizing k-nearest neighbors compared to regression algorithms and evaluated on real world data sources
Source: Sci Rep. 2026 Jan 7;16:3859. doi: 10.1038/s41598-025-33966-9 (PMC12852706; doi:10.1038/s41598-025-33966-9)
Supplement: Supplementary file 2 — Supplementary Material 2 [file 41598_2025_33966_MOESM2_ESM.docx]

**Appendices**

**Appendix A.1 (Notation Consistency)**

**Correspondence between Theoretical and Algorithmic Variables of the Arithmetic Method Algorithm (AMA)**

The variables in the practical implementation correspond directly to those in the theoretical AMA formulation. The data matrix A and vector b are represented by the arrays $a_{1\times i}$ and y; the solution $\boldsymbol{x}_{\left\{ \boldsymbol{AMA} \right\}}$ and reconstructed output A$\boldsymbol{x}_{\left\{ \boldsymbol{AMA} \right\}}$ correspond to $x_{1\times i}$ and $\hat{y}$, and the theoretical residual ‖ A$\boldsymbol{x}_{\left\{ \boldsymbol{AMA} \right\}}$ − b‖₂ is evaluated empirically by the percentage error ε. The iteration parameter m controls the problem dimension, allowing the study of numerical stability (via ε) and computational efficiency (via t). Thus, the algorithmic implementation realizes the mathematical operator $\boldsymbol{L}_{\left\{ \boldsymbol{AMA} \right\}}$​ and validates its theoretical properties of stability, bounded residual, and continuity under perturbations. Briefly, every variable in the algorithmic pseudocode has a clear mathematical counterpart: A, b, $\boldsymbol{x}_{\left\{ \boldsymbol{AMA} \right\}}$, $\hat{\boldsymbol{y}}$, ε, t, all have *direct 1-to-1 equivalence* and $\boldsymbol{L}_{\left\{ \boldsymbol{AMA} \right\}}$ and perturbation terms are *implicitly realized* through repeated algebraic operations and random initialization.

| **Theoretical Notation (Mathematical Formulation)** | **Meaning (Theory)** | **Algorithmic / Practical Variable (Implementation)** | **Explanation / Connection** |
| --- | --- | --- | --- |
| **A**$\boldsymbol{\in}\boldsymbol{R}^{\boldsymbol{m\times n}}$ | Coefficient (data) matrix in the linear system (A x = b). | $\boldsymbol{a}_{\boldsymbol{1\times i}}$ **(row of random coefficients)** | Each row $a_{1\times i}$ corresponds to the vector of coefficients ($a_{1}, a_{2}, \ldots, a_{i}$) forming the matrix A in each iteration. |
| **b**$\boldsymbol{\in}\boldsymbol{R}^{\boldsymbol{m}}$ | Target (right-hand side) vector of the linear system. | **y (actual value)** | In each iteration, y is the known value used in (Ax = b). |
| $\boldsymbol{x}_{\left\{ \boldsymbol{AMA} \right\}}\boldsymbol{\in}\boldsymbol{R}^{\boldsymbol{n}}$ | Solution vector estimated by AMA from (A x = b). | $\boldsymbol{x}_{\boldsymbol{1\times i}}$ **(vector of polynomial variables)** | Computed as  $\left( x_{j} = \frac{y}{\left( j*a_{1,j} \right)} \right)$, corresponding to the AMA algebraic operation for each component of x. |
| **ŷ = A** $\boldsymbol{x}_{\left\{ \boldsymbol{AMA} \right\}}$ | Predicted output (reconstructed target) computed from the AMA solution. | $\hat{\boldsymbol{y}}$ **(predicted value)** | In lines 26–31, $\hat{y}$is obtained as (ŷ = $\sum_{j} \left( x_{1,j}*a_{1,j} \right)$), which directly reproduces the algebraic reconstruction $Ax_{\left\{ AMA \right\}}$. |
| **A x = b** | Fundamental linear system defining the problem. | **Steps 23 and 29** | The iterative computation of x and ŷ implements the same structure as (A x = b). |
| $\boldsymbol{L}_{\left\{ \boldsymbol{AMA} \right\}}$ | Linear operator implementing the arithmetic solution ($x_{\left\{ AMA \right\}}$= $L_{\left\{ AMA \right\}}$ b). | **The entire set of algebraic steps (23 and 29)**  The algebraic operations in steps (23) and (29) together implement the linear mapping $L_{\left\{ AMA \right\}} :x_{\left\{ AMA \right\}}= L_{\left\{ AMA \right\}}b.$These two steps constitute the constructive realization of the operator in algorithmic form. | Although not explicit as a single variable, the operations constructing x and ŷ realize the functional action of $L_{\left\{ AMA \right\}}$} on b. |
| ${\boldsymbol{\vert\vert A}\boldsymbol{x}_{\left\{ \boldsymbol{AMA} \right\}}\boldsymbol{-b\vert\vert}}_{\boldsymbol{2}}$ | Residual or prediction error (stability measure). | $\boldsymbol{\varepsilon}$ | $\frac{\hat{y}-y}{y}$ |
| **‖ΔA‖_2_, ‖Δb‖_2_** | Perturbations of A and b (stability analysis). | **Random variation in** $\boldsymbol{a}_{\boldsymbol{1,j}}$ **and y** | Random values generated for a and y simulate data perturbations, testing AMA’s numerical stability. |
| **C(η_A_ + η_b_)** | Stability constant bounding perturbation effects. | **Observed convergence of ε and t to minimal values** | The empirical observation that ε and t remain small validates bounded sensitivity of AMA to input changes. |
| **m** | Number of equations (dimension parameter). | **m (input loop range, 1,…,1,000,000)** | The number of polynomial dimensions or equations tested — theoretical system size. |
| **Iteration index (i)** | Dimension index / simulation step. | **i (external loop counter)** | Corresponds to dimension growth in A (increasing number of coefficients). |
| **t** | Execution time (computational cost). | $\boldsymbol{t =}\boldsymbol{t}_{\boldsymbol{e}}\boldsymbol{-}\boldsymbol{t}_{\boldsymbol{s}}$ | Measured elapsed time of computing AMA solution — validates efficiency. |
| **I** | Index vector (record of dimensions). | **I** | Collects tested dimension sizes, used for plotting and analysis. |
| **Y** | True (target) output vector. | **Y** | Stores actual values y for each iteration (ground truth). |
| $\hat{\boldsymbol{Y}}$ | Predicted output vector. | $\hat{\boldsymbol{Y}}$ | Stores predicted values y ̂ obtained via AMA (predicted response). |
| **E or ε** | Error measure (residual magnitude). | **E (vector of ε values)** | Captures normalized residual per iteration, directly comparable to ‖A$x_{\left\{ AMA \right\}}$− b‖. |
| **T** | Execution time vector. | **T** | Collects execution times for different m, corresponding to computational performance. |
| $\boldsymbol{A}^{\boldsymbol{+}}$**,** $\boldsymbol{x}_{\left\{ \boldsymbol{LS} \right\}}$ | Reference least-squares solution. | **Not explicitly computed** | The practical AMA uses a direct algebraic inverse, implicitly approximating ($A^{+}$) without matrix factorization. |
| **Nonlinear AMA 𝒜(A,b)** | Smooth generalization of AMA with local Jacobian (J_b_ 𝒜). | **Randomized repeated calls to AMA (over 10⁶ iterations).** | Empirically validates smoothness and local stability under arbitrary data perturbations. |

**Appendix A.2 Derivation of α***

In the practical AMR algorithm, the same principle of optimal blending is realized empirically: the algorithm iterates over candidate α values in (0,1], evaluating each combination of AMA and k–NN predictions on the dataset and selecting the one that minimizes the empirical prediction error (MAE). This empirical grid-search procedure serves as a numerical approximation to the theoretical minimization of the expected mean squared error (MSE) described below. The following derivation establishes the analytic form of the MSE-optimal blending coefficient α*, which the algorithm approximates in practice through discrete search.

We differentiate R(α) with respect to α, set ∂R/∂α = 0, and we solve for α. We start with the definition of the mean–squared–error (MSE) risk of the AMR predictor:

$$R\left( \alpha\right)= E\left[ \left( \alpha U + \left( 1-\alpha\right)V - Y \right)^{2} \right]$$

where $U=ŷ_{AMA}(x)$ the prediction of the AMA model, $V=ŷ_{kNN}(x)$ the prediction of the k–NN model and Y the true target variable.

Our goal is to find the **value of α that minimizes R(α).**

$$\left( \alpha U + \left( 1-\alpha\right)V - Y \right)^{2}= \left[ V - Y + \alpha\left( U - V \right) \right]^{2}= \left( V-Y \right)^{2}+ 2\alpha\left( V-Y \right)\left( U-V \right)+ \alpha^{2}\left( U-V \right)^{2}.$$

Using linearity of expectation:

$$R\left( \alpha\right)= E\left[ \left( V-Y \right)^{2} \right]+ 2\alpha E\left[ \left( V-Y \right)\left( U-V \right) \right]+ \alpha^{2}E\left[ \left( U-V \right)^{2} \right].$$

$$\frac{\partial R\left( \alpha\right)}{\partial\alpha}=2 E\left[ \left( V-Y \right)\left( U-V \right) \right]+ 2\alpha E\left[ \left( U-V \right)^{2} \right].$$

At the minimum, $\frac{\partial R\left( \alpha\right)}{\partial\alpha}=0$ $\overset{\Rightarrow}{}$ $2 E[(V-Y)(U-V)] + 2\alpha E[\left( U-V \right)^{2}$ = 0 $\overset{\Rightarrow}{}$

$E[(V-Y)(U-V)] +\alpha E[\left( U-V \right)^{2}$ = 0 $\overset{\Rightarrow}{}$ $\alpha$ = $-\frac{E[(V-Y)(U-V)]}{E[\left( U-V \right)^{2}}$

Differentiating R(α) with respect to α and setting the derivative to zero gives the **unique minimizer (**optimal blending weight**)**:

$\alpha^{*}$ = $-\frac{E[(V-Y)(U-V)]}{E[\left( U-V \right)^{2}}$

This proves that AMR’s weighting rule is theoretically grounded: it is the MSE-optimal convex combination of the two predictors, AMA and k-NN.

Because V−Y = −(Y−V):

$\alpha^{*}$ = $\frac{E[(V-Y)(U-V)]}{E[\left( U-V \right)^{2}}$

or equivalently, in terms of covariance and variance:

$$\alpha^{*}=\frac{Cov\left( U-V, Y-V \right)}{Var\left( U-V \right)}$$

Because always $E\left[ \left( U-V \right)^{2} \right]>0$ (except the case where (U=V)), so this **α*** is **unique**. The **α*** is the **mean–squared–error optimal blending coefficient** for combining AMA and k-NN predictions.

The AMR algorithm introduces an additional tuning parameter δ, which controls the definition of neighborhood proximity in k–NN. This parameter indirectly influences the optimal value of α by modifying the variance and bias properties of the k–NN estimator V. Hence, δ can be interpreted as a practical mechanism for adjusting the bias–variance balance that α* theoretically optimizes.

In practice, the expectations in this expression are unknown and must be estimated from finite samples. An unbiased sample estimator of α* is obtained by replacing expectations with empirical averages:

$$\hat{\alpha}= \frac{\Sigma_{i}\left( y_{i}-v_{i} \right)\left( u_{i}-v_{i} \right)}{\Sigma_{i}\left( u_{i}-v_{i} \right)^{2}}$$

The AMR algorithm does not compute this value directly but instead approximates it by evaluating α over a discrete grid and selecting the value α_op_ that minimizes the empirical prediction loss (MAE). When the grid resolution is fine, α_op_ ≈ $\hat{\alpha}$, meaning the algorithm’s empirically optimal blending coefficient is a numerical approximation to the theoretical MSE-optimal value. In this sense, the empirical procedure implemented in AMR acts as a *sample-based discretization* of the analytic estimator α̂, translating the continuous optimization of R(α) into a finite grid evaluation guided by observed data.

While the theoretical derivation minimizes the expected MSE, the algorithm employs MAE as the optimization criterion. Both MAE and MSE are convex loss functions that measure average prediction deviation, and minimizing either leads to similar optimal weighting behavior. However, MAE is less sensitive to outliers and offers greater numerical stability in empirical optimization. Therefore, the use of MAE in the algorithm can be regarded as a robust empirical adaptation of the theoretical MSE objective.

**Appendix A.3 (Notation Consistency)**

**Correspondence between Theoretical and Algorithmic Method Regression (AMR) Variables**

The following table provides a detailed correspondence between the mathematical symbols used in the theoretical framework of the Arithmetic Method Regression (AMR) and the variables employed in its algorithmic implementation.
Each theoretical variable is explicitly mapped to its computational counterpart appearing in the pseudocode of the Arithmetic Method Regression (AMR) algorithm (Table 2). Where relevant, short justifications are provided to demonstrate how each algorithmic operation reflects the mathematical model.

| **Theoretical Symbol** | **Meaning (Theory)** | **Algorithmic Variable** | **Meaning (Implementation)** | **Connection / Notes** |
| --- | --- | --- | --- | --- |
| $\boldsymbol{D}_{\boldsymbol{n\times m}}$ | Input dataset of n samples and m features | $\boldsymbol{D}_{\boldsymbol{n\times m}}$ | Input dataset | Direct correspondence. |
| $\boldsymbol{A \in}\boldsymbol{R}^{\boldsymbol{m\times n}}$ | Data (predictor) matrix | $\boldsymbol{A}_{\left( \boldsymbol{n-1} \right)\boldsymbol{\times}\left( \boldsymbol{m-1} \right)}^{\boldsymbol{mo}}$ | Model matrix returned by model.function() | Represents internal regression model of AMA within AMR loop. |
| $\boldsymbol{b \in}\boldsymbol{R}^{\boldsymbol{m}}$ | Response (dependent variable) vector | $\boldsymbol{Y}_{\left( \boldsymbol{n-1} \right)\boldsymbol{\times1}}^{\boldsymbol{tr}}$ | Training response vector | Used to compute AMA model coefficients. |
| $\boldsymbol{x}_{\boldsymbol{AMA}}$ | AMA solution vector | $\left( \begin{aligned} \boldsymbol{implicit inside} \\ \boldsymbol{model.function}\left( \right) \end{aligned} \right)$ | Internal coefficients of AMA model | The algorithm uses AMA as a sub-routine. |
| $\boldsymbol{ŷ}_{\boldsymbol{AMA}}$ | AMA predicted output | ${\hat{\boldsymbol{y}}}_{\boldsymbol{AMA}}^{\boldsymbol{pr}}$ | Predicted value from AMA | Combined in convex blend. |
| $\boldsymbol{ŷ}_{\boldsymbol{kNN}}$ | k-NN predicted output | ${\hat{\boldsymbol{y}}}_{\boldsymbol{kNN}}^{\boldsymbol{pr}}$ | Predicted value from k-NN | Combined in convex blend. |
| $\boldsymbol{ŷ}_{\boldsymbol{AMR}}$ | Combined AMR prediction | ${\hat{\boldsymbol{y}}}^{\boldsymbol{pr}}$ | Overall AMR predicted value | Implemented as ${\hat{\boldsymbol{y}}}^{\boldsymbol{pr}}\boldsymbol{\leftarrow\alpha\cdot}{\hat{\boldsymbol{y}}}_{\boldsymbol{AMA}}^{\boldsymbol{pr}}\boldsymbol{+ \beta\cdot}{\hat{\boldsymbol{y}}}_{\boldsymbol{kNN}}^{\boldsymbol{pr}}$  . |
| $\boldsymbol{Y}$ | True response variable | $\boldsymbol{y}^{\boldsymbol{ac}}, \boldsymbol{Y}^{\boldsymbol{ac}}$ | Actual observed outputs | Used for error computation. |
| $\boldsymbol{\alpha}$ | Convex weight for AMA | $\boldsymbol{alpha}$ | AMA contribution ratio | Identical role in theory and code. |
| $\boldsymbol{\beta= 1 - \alpha}$ | Complementary weight for k-NN | $\boldsymbol{beta}$ | k-NN contribution ratio | Enforced at line 16: beta ← 1 − alpha. |
| $\boldsymbol{\alpha}^{\boldsymbol{*}}$ | Theoretical MSE-optimal coefficient | $\boldsymbol{alph}\boldsymbol{a}_{\boldsymbol{op}}$ | Empirically optimal α minimizing MAE | Discrete approximation of α*. |
| $\hat{\boldsymbol{\alpha}}$ | Unbiased sample estimator of α* | **(approximated by**  $\boldsymbol{alph}\boldsymbol{a}_{\boldsymbol{op}}\boldsymbol{)}$ | – | Finite-sample equivalent. |
| $\boldsymbol{R(\alpha)}$ | Expected MSE risk | $\boldsymbol{MAE}, \boldsymbol{MSE}, \boldsymbol{RMSE}$ | Empirical loss functions | Practical proxies for theoretical risk. |
| $\boldsymbol{\delta}$ | Neighborhood calibration parameter | $\boldsymbol{delta}$ | Heuristic radius extension for k-NN | Controls bias–variance of k-NN part. |
| $\boldsymbol{\delta}_{\boldsymbol{op}}$ | Optimal neighborhood parameter | $\boldsymbol{delt}\boldsymbol{a}_{\boldsymbol{op}}$ | Best δ minimizing MAE jointly with α | Adjusts local smoothness. |
| $\boldsymbol{k}$ | Number of nearest neighbors | $\boldsymbol{k}$ | Current number of neighbors in k-NN | Tuned jointly with δ. |
| $\boldsymbol{k}_{\boldsymbol{op}}$ | Optimal number of neighbors | $\boldsymbol{k}_{\boldsymbol{op}}$ | Best-performing k | Stored when MAE ≤ MAE_op. |
| $\boldsymbol{R}^{\boldsymbol{2}}\boldsymbol{, MSE, MAE,}$  $\boldsymbol{RMSE}$ | Performance metrics | $\boldsymbol{R}^{\boldsymbol{2}}, \boldsymbol{MSE}, \boldsymbol{MAE}, \boldsymbol{RMSE}$ | Computed using library functions | Empirical validation metrics. |
| $\boldsymbol{ET}$ | Execution time (runtime) | $\boldsymbol{ET}$ | Computed as $t_{end}- t_{start}$ | Measures computational efficiency. |
